# "All Sorts and Conditions" of Women

**Published:** 1889-02-09

**Authors:** 


### The Doctor's Pulpit.

### "ALL SORTS AND CONDITIONS" OF WOMEN.

### The Worst Wife.

A few weeks ago there was announced one of the most eccentric wills which ever appeared in the probate columns of a newspaper. An old gentleman by his last testament directed that his wife, as her share of his estate, should receive one farthing, and that the amount should be forwarded to her in an unpaid letter. It was explained that the lady had called her husband to his face "an old pig," and that her irate spouse had thus marked his sense of the indignity put upon him by his other half. It is difficult, if not impossible, to believe that for one single offence so stern and implacable a punishment should have been inflicted, even by a bad husband. The possibility is that the offensive expression used by the wife was but an index to a series of unworthy speeches and acts, dictated by ill-controlled feelings, which had in course of years succeeded in alienating the heart of the husband, and inducing a bitterness of spirit which not even death itself could soften down. It is not necessary to assume that the husband was good in order to justify a severe censure of the wife's conduct. Hardly any kind of behaviour on a man's part could bring a really good woman into such a condition of mind as would prompt her in serious earnest to call an aged man, and that man her husband, by so needlessly disgusting and offensive an epithet as "an old pig." Perhaps the appellation was a fairly correct description of him; but even granting that, it was not in any sense the kind of language which should have befouled the mouth of a wife.

Both men and women can fall low in morals and conduct; but there always seems to be a deeper depth about the degradation of a woman than is possible in a fallen man. The spirit of the typical woman is finer, more delicate, more purely pure, and more gentle than that of man. Where the bloom is off it, the loss seems more marked and more injurious. It is often held, too, that woman, being less robust in mind than man, falls more hopelessly and unrestrainedly than he does. When once she has completely given way to baseness of life, her self-abandonment is held to be more complete and final, But can a woman give way to baseness and still continue to be a wedded wife? Undoubtedly! There are women whose outward virtue is unimpeached and unimpeachable, but whose mind and heart are as thoroughly worthless and as dead to all true womanhood as the entire want of a moral sense can make them.

What constitutes a bad wife? Disloyalty: an alienated heart. It is not necessary for a married woman to be a flirt or an unfaithful wife in order to be disloyal and base in spirit. That the married flirt is contemptible goes without saying: she is not worthy even of discussion. Her head is as destitute of sense as her heart is of worth. If she have children, it would be well for them, as for her husband, if she were shut up in a lunatic asylum or tossed into the horse-pond and drowned. Of course, we are not advising either of these measures; we are simply giving utterance to the mood of contempt which is produced in the mind by the unreasoning behaviour of what ought to be a reasonable creature. It may be natural for women to love admiration. But children love port wine; is it therefore right that they should be allowed to poison themselves with it? A woman may have a dull and unappreciative husband. But even supposing that very little regard is owed to such a husband, does she owe no regard to herself, none to her children, none to her parents and her family? How base and worthless is the married flirt! She is but a step removed from the woman who is false and unfaithful.

But a wife need not be either a flirt or unfaithful in order to qualify herself to rank with the worst. All that she needs to do is to give herself up to a life of determined selfishness. Let her once allow herself to think that the world is a place in which the principal business is personal enjoyment; that the chief value of a husband consists in his power to supply her with the means of pleasure; that children are not desirable because they are inconvenient and exacting; that home is dull when it is without guests, and that life is only worth living when spent in excitement and vanity: when she has arrived at this level she does not need to sink any lower in order to be one of the most worthless of wives. An unfaithful woman or a confirmed flirt is not a wife at all. The woman whose heart is unmitigated selfishness may be a wife, but she is neither gold, silver, nor even bronze, but only a signed and filled-up cheque marked, "no effects."

There is a whole dead sea of concentrated bitterness in many married men's hearts. The miserable vanity and reckless selfishness of their wives have been yielded to so often that the spectre of ruin has been raised at last. Many a naturally honest man has been driven first to craft, and then to theft, and then to destruction by a wife to whom he could not say "No." A large part of the financial pressure of the present times is due to the social struggling of women and the expenditure it involves. What does a man care whether his neighbour Jones, who formerly lived in a smaller house, now lives in a larger one than he himself occupies? Or whether Mrs. Browne has got her drawing-room newly furnished in the most modern and extravagant way? If Jones can afford a big house, by all means let him have it; and if new chairs and carpets and curtains will make the small soul of Mrs. Browne happy, why grudge her such happiness? A sensible husband does not trouble himself to think twice on such a subject; but his indescribable wife may make herself and her whole household utterly miserable about it. Every time she passes Jones's house she thinks Mrs. Jones is looking out of her window with an expression of mingled triumph and pity on her face; every time Mrs. Browne calls upon her she thinks that lady is mentally contrasting the old-fashioned chairs and faded carpets of the drawing-room with her own brand new and latest-fashion glories. Alas! what a world it is! Sense and social salvation are synonymous silliness, as of a sheep, and imbecility, as of an idiot fill the world, and bring it to misery and ruin.

Some people ask what use it is to speak in this way? Do we expect to make foolish women any wiser, or weak-willed men any stronger? Undoubtedly! There is in almost every man and woman a capacity for improvement. There is no better established fact of science than this. It is every teacher's duty, whether he be an expounder of those parts of physiological and medical science that have a general bearing upon the public well-being, or of any other science or faith, first of all to make his readers see and understand what it is which they do wrong, and what it is of right which they leave undone, but ought to do. The doctor in his daily rounds sees whole worlds of misery created and carried on by those who insist upon competing with their neighbours in extravagance and display. He sees wives growing more and more hopelessly frivolous; daughters brought up without the smallest modicum of real sense and understanding; husbands, staggering, stumbling, falling, and dying of despair at their powerlessness to keep abreast of those whose resources are larger than their own. What does it all end in? Collapse and ruin! Women have it in their power to regenerate and make wholesome and happy the whole social system. That is why, in this place, they are so frequently appealed to on all sides of their intellect and heart.

*(To be continued.)*

